# An exploratory study of the barriers and facilitators to the implementation of community health worker programmes in conflict-affected South Sudan

**DOI:** 10.1186/s13031-021-00422-0

**Published:** 2021-11-18

**Authors:** George William Lutwama, Maryse Kok, Eelco Jacobs

**Affiliations:** 1 Health Pooled Fund, American Embassy Residency Road, Juba, South Sudan; 2 KIT Health, Royal Tropical Institute, Mauritskade 63, Amsterdam, 1090 HA The Netherlands

**Keywords:** Boma health initiative, Boma health worker, Conflict-affected, Community health worker, Health pooled fund, South Sudan

## Abstract

**Background:**

Community health workers (CHWs) are crucial for increasing access to health services to communities. Due to decades of conflict and under-funding, access to health care in South Sudan remains severely limited. To improve equitable access to healthcare, the government has introduced “the Boma Health Initiative (BHI)”, a strategy to harmonise community health programmes across the country. In order to scale up the BHI, it is necessary to assess the recent CHW programmes and draw lessons for future implementation. This study aimed to explore the characteristics, barriers, and facilitators to the implementation of CHW interventions in South Sudan between 2011 and 2019.

**Methods:**

The study used a qualitative approach drawing from 26 key informant interviews and a scoping review of 21 Health Pooled Fund (HPF) programme reports from October 2016 to June 2018 and policy documents from 2011 to 2019. The results were thematically analysed based on a conceptual framework on factors influencing the performance of CHWs.

**Results:**

Funding of CHW programmes has come from international donors, channelled through non-governmental organisations (NGOs) that have implemented a variety of CHW programmes. Communities have been participating in the selection of voluntary CHWs, intervention areas, and occasionally in the supervision of activities performed by CHWs. The coordination mechanisms among stakeholders have been weak, leading to wastage and duplication of resources. Although training of CHWs is done, training duration was short, and refresher-trainings were rare. There were and still are disparities in the type of incentives provided to CHWs. Monitoring and supportive supervision activities have been insufficient; drug misuse and stock-outs were common.

**Conclusion:**

Despite their challenges, CHW programmes can be implemented in conflict-affected South Sudan if the local human capital is leveraged and engaged by NGOs as implementing partners. Robust coordination efforts are required to build synergies among stakeholders for the effective implementation of the BHI strategy.

## Background

South Sudan has experienced nearly continuous violent conflict since 1955 [1, 2] and has some of the worst health indicators in the world. The maternal mortality ratio is estimated to be at 1,150 deaths per 100,000 live births, child mortality at 99 per 1,000 live births, and infant mortality at 65 per 1,000 live births, which can be mostly attributed to preventable causes [3]. The conflict, which has flared up again since 2013, has led to widespread looting and destruction of health facilities. There have been massive displacements of citizens, mostly children and women, to remote areas where basic health services are scarcely available [1, 3]. It is estimated that over 400,000 lives have been lost since 2013 [4]. The health service coverage remains low, and access to care is severely hampered, particularly for rural communities [5].

With government financing for the health sector at less than 4%—far below the 15% commitment under the Abuja declaration [6, 7], nearly 80% of the health services are provided by non-governmental organisations (NGOs) [3, 8]. Since 2012, a basic package of health and nutrition services [9] is provided across all states through contracted NGOs via pooled donor funding such as the South Sudan Health Pooled Fund (HPF) and the World Bank [1, 3]. HPF is a multi-donor funding mechanism that consists of six donors: the United Kingdom, Canada, the European Union, Sweden, the United States, and Gavi. This funding mechanism operates in 21 geographic areas in eight of the ten states and uses Ministry of Health (MoH) facilities and health workers. Currently, HPF is in the third phase, which runs until July 2023. This phase focuses on provision of healthcare services at the health facility level and expanded community health services [5, 10].

There is a huge variety in the nomenclature and classification of community health workers (CHWs) worldwide. Broadly, CHWs can be defined as health workers based in communities, who are either paid or volunteers, who are not professionals, and who have had fewer than two years of training [11, 12]. Studies from low- and middle-income countries suggest that CHW programmes can be effective in improving access to care for underserved communities [13, 14]. Following the 2018 Astana declaration on primary health care, there is renewed political commitment and leadership from the United Nations (UN) member states and global organisations to develop people-centred primary health care, building on the principles, gains, and lessons from the Alma Ata Declaration [15].

The South Sudan MoH recognises the importance of CHWs in improving access to healthcare. This is reflected in the National Health Policy 2016–2025 [16] and the National Health Sector Strategic Plan 2017–2022 [17], which place emphasis on the establishment of the community health system as a formal structure of the national health system. The community health system includes primary care provided by CHWs, Primary Health Care Units (PHCUs) and Primary Health Care Centres. Secondary care includes the County Hospitals and State Hospitals, and tertiary care is provided at national teaching, specialist, and referral hospitals [17, 18].

Before independence, Southern Sudan had a paid cadre of CHWs who were trained for nine months and who were based at the PHCU level, thus focusing on facility-based, often curative tasks. In 2007, the MoH stopped this training and focused on training nurses and midwives, while—with assistance of NGO—focusing on expanding the pool of voluntary CHWs at community level. Since then, CHW programmes in South Sudan have been uncoordinated, and largely led by humanitarian and development partners with an orientation to specific diseases [19, 20]. In response to the lack of coordination, the MoH launched the Boma^1^ Health Initiative (BHI) strategy in March 2017 with the stated goal of striving towards improved access and health coverage [19, 20]. The BHI intends to establish trained, full-time, salaried CHWs also known as Boma health workers (BHW) to promote community participation in increasing ownership and sustainability of health services [19, 20].

The BHI furthermore intends to bridge the gap between health facilities and communities through standardisation of the service packages offered by CHWs, and harmonisation of incentives across the community health programmes [20]. The BHI focuses on child health, safe motherhood, control of communicable and non-communicable diseases, disease surveillance, and reporting on service delivery and vital statistics [19, 20]. It is estimated that approximately 44% of the population in South Sudan live within reach of health facilities and have consistent access to primary care services [10, 20]. Moreover, service provision at facility level repeatedly deteriorates during conflicts [21]. In some locations, community-based programmes discontinued due to active mine laying or presence of explosive remnants of the war [1]. Given the BHI’s aim to scale up community health in South Sudan it is imperative to draw lessons from previous CHW programmes and assess them alongside evidence from community health in comparable settings. The aim of this study was to explore the characteristics, barriers, and facilitators to implementation of recent CHW programmes in the states supported by the HPF programme in South Sudan, in order to inform further scale-up of the BHI, and the design and implementation of CHW programmes in other low resource, conflict-affected settings.

## Methods

### Study design

The study followed a qualitative approach using key informant (KI) interviews and a review of the HPF programme reports from October 2016 to June 2018 and policy documents from 2011 to 2019. The policy documents included the Basic Package of Health and Nutrition Services for South Sudan (2011) [9], the South Sudan Health Sector Development Plan, (2011- 2015) [22], the Ministry of Health Policy Framework (2013–2016) [23], the National Health Policy (2016–2025) [16], the National Health Sector Strategic Plan (2017–2022) [17], the Boma Health Initiative Costing and Investment Case Analysis (2019) [19], and the Boma Health Initiative Strategy (2016) [20].

### Study setting

The study was carried out at the request of HPF and therefore focused on eight of the ten states, where health service delivery is supported by their programme. At the Boma level, the services are delivered by CHWs or a primary health care unit. The primary health care centre is located at payam^2^ level, and the hospital at county or state level [24]. At the time of the study, the HPF supported primary health care services in 794 health facilities and the initial roll out of the BHI in 23 of 55 counties from six of the eight states.

### Participants

The participants were purposefully selected from the MoH, HPF, NGOs, and UN Agencies, based on their current or previous experience in the health sector and community health programmes. This enabled gathering a wide range of perspectives from policy, management, and practice. CHWs were not included as participants because of budget and security-related constraints. The end of project reports from the NGOs contracted to implement the second phase of the HPF programme (2016–2018) were also reviewed.

### Data collection and analysis

We reviewed the twenty HPF implementing NGOs’ reports and relevant policy documents between June and August 2019. For document analysis, a list of compiled materials was annotated in Microsoft Excel describing the source, date, and data. The primary author reviewed the materials for information relevant to community health and summarized data into a spreadsheet. The review focused on the characteristics of community health programmes, types of CHWs, types of services offered, challenges faced, and lessons learnt during the implementation. The findings from this review fed into the discussions with the study participants.

We conducted twenty-six interviews in English between 21st August and 19th September 2019 using a semi-structured interview guide. Seventeen interviews were face-to-face, three via Skype and six by telephone. On average, the interviews lasted 69 min, ranging from 48 to 90 min. Table 1 shows the characteristics of the participants.

**Table 1:** Characteristics of the participants

Item	Category	Number (N = 26)
Gender	Women Men	07 19
Organisation	Non-Government Ministry of Health (National and State) United Nation Agencies	13 10 03
Roles in Organisation	Coordination and Policy Programme management Programme implementation	08 07 11

Fifteen participants consented to audio recording of the interviews. The recorded interviews were transcribed verbatim. From the non-recorded interviews, comprehensive notes were taken, and transcripts were finalised thereafter. All interview transcripts were imported into NVivo 12 software (QSR International) and thematically analysed. The coding was done based on predetermined themes adapted from a conceptual framework on factors influencing performance of CHWs in low- and middle-income countries [25]. This framework identified three major categories of factors that could influence the CHW programmes: intervention design, health systems and contextual factors. Intervention design factors consist of human resources management (such as recruitment, training, supervision, remunerations etc.), quality assurance, tasks and time spent on delivering, co-ordination and communication between the professional health workers and CHWs, community involvement, and logistics and resources. Health system factors involve governance and coordination structures, funding mechanisms, health service delivery, human resources provisions, health information systems, as well as supplies and logistics. The contextual factors are socio-cultural, economic, environment, political (including health policies) as well as safety and security.

### Ethical considerations

Ethical approval was obtained from the research ethics committee of KIT Royal Tropical Institute, Amsterdam, and the South Sudan Ministry of Health research ethics review board. Informed written consent (for face-to-face) and verbal consent (for Skype and telephone interviews) were obtained from the participants, their privacy and confidentiality were assured and respected during and after the interviews. No financial incentives were provided for participation in the study. The HPF senior leadership granted permission to access the programme reports and did not interfere in any way with the research process.

## Results

### Characteristics of community health worker programmes

While the community level health structure was provided for in the basic package of health and nutrition services for South Sudan (2011), the Ministry of Health Policy Framework (2013–2016) and the Health Sector Development Plan (2011–2015) provided limited guidance on how to realise it and put more emphasis on curative health services delivery. However, under the current health policy (2016–2026) and the National Health Sector Strategic Plan (2017–2022), the government is committed to establishing the community health structures (Payam Heath Committees, Boma Health Committees (BHC) and Boma Health Teams (includes CHWs)) under the BHI [9, 16, 17, 20, 22, 23].

Study participants reported that this expanded focus on community health at policy level translated into a variety of CHW programmes in practice. These programmes included integrated community case management (iCCM)^3^; immunisation outreaches; water, sanitation, and hygiene promotion; neglected tropical diseases (NTD) (e.g., preventive chemotherapy and guinea worm disease surveillance); HIV/AIDS behaviour change communication; pregnancy-related services, referrals of patients; and screening for malnutrition.

The interviews and information from the HPF reports revealed that programme activities were mostly in the primary care facilities. To a lesser extent, the implementing NGOs used community-led approaches to increase demand for primary care services. These approaches included the forming and the training of health facility and Boma Health Committees to provide oversight in implementing the primary care activities. Other approaches were community mobilisation and promotion by voluntary CHWs, mother-to-mother support groups, and school health clubs; the sourcing of local materials to renovate health facilities; the distribution of misoprostol to prevent postpartum haemorrhage and using CHWs to treat specific diseases of children under five years of age.

### Intervention design factors

#### Human resources management

##### Characteristics of community health workers

Since the CHWs programmes in South Sudan were mostly partner-led or disease specific [20, 22], there were variations in the characteristics of CHWs, the tasks they performed, how they were recruited, trained, supervised, incentivised, and how they reported on their activities. The description of the types and characteristics of the CHWs (all volunteers) that worked under the community health programmes are presented in Table 2.

**Table 2:** Types and characteristics of the voluntary community health workers

Types of CHWs**	Programme specific CHW worked for	Specific tasks performed by CHWs	Duration of training sessions	Gender prerequisites	Types of incentives
Community based distributors (CBDs)	Integrated community case management (iCCM) and Community case management (CCM)	Treatment of children under five for pneumonia, malaria, diarrhoea, health education on child health, and screening for malnutrition and referrals	Six days initial training and three days refresher training	Female and male	Material/in-kind incentives like soap, sugar, raincoats, gumboots, t-shirts etc. Some implementing NGOs gave monetary incentive ranging from USD 25 -50
Community Based Distributors (CBD) supervisors	Integrated community case management (iCCM) and Community case management (CCM)	Supervision of CBDs, compiling and submitting reports and ensuring that CBDs conduct effective treatment of children under five in the community	Six days initial training as CBD plus two to three additional training days as supervisors	Female and male	Monetary incentives (USD 100) and materials incentives like raincoats, bicycles, gumboots, t-shirts, backpacks etc
Community drug distributors (CDDs) and CDD supervisors	Neglected tropical diseases (NTD)	NTD drugs distribution, community mobilisation, awareness raising, and referral of suspected NTD cases to the health facilities Supervisors monitor activities of the CDDs and ensure reporting on the interventions	One to three days training depending on the intervention and one to two days refresher training. Supervisors received additional two days training	Female and male	Material incentives/in-kind incentives like soap, sugar, t-shirts, and transport refund of USD 5–10 when they attend meetings or trainings. In some cases, bicycles were given to the supervisors
Community nutritional volunteers (CNVs)	Nutrition	Screening for malnutrition, nutrition education and referral of malnutrition cases from community to health facilities, and follow-up of children discharged	Three to four days training and two days refresher training	Female and male	Performance based monetary incentive of USD 30–35 monthly and material incentives like soap and sugar, and t-shirts, gumboots, raincoats etc
Home Health Promoters (HHPs)	Maternal and child health and Water, Sanitation and Hygiene	Health education, distribution of misoprostol for prevention postpartum haemorrhage, mobilising for health campaigns	Two to ten days training depending on the intervention and two to three days refresher training	Female and male	Material/in-kind incentives like soap and sugar, t-shirts, gumboots, raincoats, torches etc
Traditional Birth Attendants (TBAs)	Maternal health	Some community deliveries, referral of mothers for skilled birth attendance, health education	Seven to fourteen days training and two to three days refresher training	Female	Material/in-kind incentives such as soap, sugar, gloves, delivery kits, mama kits, torches etc
Mother-to-mother support groups (M2MSG)	Nutrition and MNCH	Support mothers and their children under five on healthy living, follow on immunisation status of children and antenatal care services	Two to three days training depending on the intervention and one to two days refresher training	Female	Material/in-kind incentives soap, sugar, t-shirts, caps etc.,

**All voluntary CHWs worked on part-time basis

The community health programmes were implemented in the Counties and Bomas, however, in some cases, it was not uncommon to find similar CHW programmes in the same counties but funded and implemented by different partners and giving different types of incentives. At times unrelated CHW programmes in the same location used the same voluntary CHWs for their programmes.

##### Selection and recruitment of community health workers

According to guidance under the BHI, CHWs should be chosen from the communities they will serve, and communities should participate in their selection to secure the community’s trust and ownership of the programme [20]. Supported by the implementing NGOs and the County Health Department (CHD), the community leaders and the BHCs led the selection of CHWs. For nearly all the CHW programmes, the selection criteria included being a resident of the area, ability to read and write, being trainable, aged between 18 to 45 years, willing to work as a volunteer, well behaved, and being approved by the community. The participants revealed that in some areas there was favouritism during the recruitment process while in others, it was hard to find literate people. “There are some communities where people selected were not living in those particular Bomas, they (leaders) instead selected people from towns” (KI12, Man, MoH). A few participants mentioned that there was preference for older women as CHWs, since they were more stable in the community than men and younger people, nonetheless they are limited by their education levels. Some participants proposed that the current primary level education requirements for the selection of CHWs to be relaxed for women to ensure gender equality.

##### Training of community health workers

As recommended by WHO, the South Sudan National Health Policy (2016–2026) mandates short-term training approaches for CHWs in specific skills to perform specific tasks under supervision [16]. The study participants indeed indicated that CHWs received training and occasionally refresher training sessions. The participants reported variations in the duration of the training sessions, ranging from one day to (before independence) nine months.^4^ For iCCM, the participants reported that the duration of the initial training was six days, and refresher training sessions took three days. With other programmes like on NTDs, the duration of training sessions was one day for the community drug distributors, two days for the supervisors, and five days for the training of trainers.

The participants reported that differences in the duration of training sessions (for the same programmes) across the counties and the implementing NGOs were due to insufficient budget to carry out such activities. For instance, training of home health promoters used to take two or three days in some counties while it took five days in others. The participants mentioned that the refresher training sessions were irregular, either due to lack of funds or the neglect by the implementing NGOs. Many CHWs did not have the required education and did not get a chance to practice under the supervision of the linking health facility before their deployment into the community. With little formal education some CHWs found it difficult to understand the training materials, the reporting tools, and interpretation of key health education messages.

The participants cited the use of simple pictorial training materials to facilitate learning. The participants also mentioned that unlike the training materials for iCCM and NTD programmes, the current BHI training materials are bulky, heavily worded, and lack pictographic aspects, which makes it difficult for the low educated CHWs to understand. The participants recommended that after training, the CHWs should be attached to the nearby health facility to have supervised community practice to acquire skills and hands-on experience.

Most participants indicated that iCCM and NTD programmes have training curricula developed by the MoH, with support from partners, in particular UNICEF and WHO.

The training programmes for the home health promoters, the BHCs, and the traditional birth attendants, did not have curricula. In this case, the NGOs developed their training materials, which led to variations in the content and the duration of the training sessions. “Under the South Sudan health transformation project in Central and Western Equatoria, we (NGO) developed the training manuals for the village health committees, the home health promoters, and the traditional birth attendants in consultation with the State Ministries of Health” (KI19, Man, UN).

##### Supervision

Policy documents in South Sudan point to the need of regular monitoring and supportive supervision of CHWs [20, 23]. They furthermore outline that monitoring and supportive supervision should be performed using specified tools and should be documented by supervisors. It is important that the community should be part of the main supervisory team of CHWs [17, 18]. The supervision must be constructive, useful and provide guidance to the implementers of the programme. In South Sudan, full responsibility for the supervision of health facilities and CHWs has been shifted to county levels [17, 20]. From the participants and the HPF reports, it was found that the BHC members and the local community leaders supervised the activities of the CHWs. Other supervisors were community-based supervisors (for the iCCM and NTD programmes), the NGO technical staff, the CHD staff, and occasionally staff from the nearby health facilities.

The participants declared that supervision of CHWs was through monthly joint supervision visits. A few participants mentioned that they used group meetings with the CHWs to monitor performance. The participants cited those inadequate and infrequent supervision visits were partly due to insecurity, lack of transport, and poor road networks in the counties. For iCCM, there was limited involvement of the CHD in supervising CHWs. Moreover, the nearby health facility staff were not involved in the supervision of community activities, leading to gaps between the community and health facility activities. The participants cited the lack of payam health departments and the occasional dormancy of the BHCs as obstacles to the supervision of CHWs.

##### Performance appraisal

Nearly half of the participants mentioned that performance assessment of CHWs was done by comparing CHWs’ achievements against the planned activities. Some of the indicators used were the number of patients treated, the number of reports submitted, daily attendances, and the number of community mobilisation sessions conducted. A few participants mentioned that in previous CHW programmes, there was no structured performance assessment framework for CHWs. “Performance management of CHWs is something we (policy makers) have to look into critically as we start Boma health initiative implementation.” (KI25, Woman, UN).

##### Incentives

Until recently, South Sudan did not have a harmonised structure for incentivising CHWs. The BHI strategy mandates that the CHWs should be paid a salary from the government and the, implementing partners should give additional incentives [20]. The participants mentioned that they offered CHWs both material and financial incentives from NGOs depending on the programme. Some of the CHWs (e.g., CBDs), received monetary incentives ranging between five to 100 United States dollars per month. In some cases, such as NTD and maternal child health programmes, transport refunds were offered to CHWs when they attended training and when they accompanied mothers with danger signs to the health facilities.

Some participants mentioned that they provided only material incentives, e.g., soap, sugar, gumboots, raincoats, torches, umbrella, t-shirts, and bicycles, while others provided only training and supervision for motivation. “We (NTD programme) have two layers of volunteers, the ones at the village level, we only train and train…., and keep supervising them, but no other incentive is given…” (KI02, Man, MoH). Nearly all participants mentioned that there are no separate incentives given to CHWs for referrals since this was part of their routine work.

#### Quality assurance

##### Monitoring and evaluation

For the iCCM and the NTD programmes, the CHWs used pictorial registers and summary forms to collect and report data. The Boma supervisors collected the CHW reports and submitted them to the supporting NGO or CHD offices. At the CHDs, the reports were checked for completeness and entered into the district health information system. Some participants noted that while the CHWs and the supervisors submitted reports, the data quality was questionable. Most participants also reported that the current BHI reporting tools are complex and are difficult to complete for the low educated CHWs.

#### Community and health system links

The National Health Policy (2016–2025), the Heath Sector Development Plan (2012–2016) and the National Health Strategic Plan (2017–2022) emphasise that communities should participate in health services delivery, health promotion, disease prevention activities and health governance, to enable them taking charge of improving health [16, 17, 22]. The participants reaffirmed that community leaders are involved in several stages of implementation of CHW programmes. The community leaders facilitated the situation analysis, mapping of communities, selection of locations for implementation, selection of volunteers, community mobilisation and awareness, and supervision of CHW activities. The participants acknowledged the relevancy of community sensitisation meetings in programme implementation. These meetings resulted in people having the same understanding of the programme activities and what each party involved will contribute.

In some counties, the implementing NGOs prepositioned the CHWs’ medical commodities at the nearby health facilities to facilitate the monitoring of and the reporting on drug usage. The health facilities also served as replenishment points of the CHW stock. In other counties, however, the NGOs supplied the medical commodities directly to the CHWs without passing through the CHD or the nearby health facility. The participants indicated that the understaffing and heavy workloads limited the health facility staff’s ability to supervise CHW activities.

##### Communication and coordination

The Health Sector Development Plan (2012–2016) mentions poor coordination between the MoH, State Ministries of Health and NGOs involved in health care delivery as a key challenge [22]. The participants cited the importance of quarterly review meetings held by NGOs at the county level as good avenues for disseminating information to and getting feedback from communities. Some BHCs also provided feedback from the communities to health facilities or the CHD, and vice versa. Furthermore, the participants proposed the formation or reactivation and strengthening of BHCs to provide oversight roles in the implementation of community health activities. They stressed that strong coordination and regular information sharing among stakeholders can eliminate duplication of services.

#### Resources and logistics

##### Supplies

CHWs kept the medical commodities, like antimalarial drugs, in metallic boxes at their homes. For the iCCM programme, the NGOs procured and distributed medical commodities to the CHWs without passing through the MoH. In the NTD programme, the donors procured the drugs, but the distribution was mostly through the MoH supply chain. The participants reported stock-out of some drugs and commodities resulting from misuse, for example, drugs given to ineligible clients. The participants also mentioned that in some counties, the storage facilities were inadequate, which made it difficult to preposition drugs during adverse weather conditions.

##### Job aids

The participants mentioned that CHWs were supplied with some job aids to facilitate their work. For example, under iCCM, the CHWs had timers, beads, mid-upper arm circumference tapes, and pictorial charts to support health education and the assessment and treatment of children. Some participants mentioned that the use of beads and timers by the CBDs without much formal education helped to improve the quality of diagnosis and management of pneumonia among the children under five years of age.

##### Transport

The CHWs moved on foot when they were conducting their activities. In the NTD and iCCM programmes, the CHW supervisors were given bicycles to facilitate their movements in the community. At the county and NGO level, a motorcycle or vehicle was allocated to support the supervision and distribution of commodities.

### Health systems factors

#### Governance of community health worker programmes

As stated in the South Sudan National Development Plan 2012–2016 [22], at the national level, the MoH provides overall leadership; develops policies, guidelines, and standards; engages in advocacy and resource mobilisation and supervises the overall health care service delivery in the country. Although the MoH provided oversight over the CHW programmes, most of the coordination, as stated by study participants, was by donors and the contracted NGOs. One participant mentioned that before 2014, the iCCM programme was not embedded into the MoH health system, and had no funds allocated from the national budget. Other participants mentioned that technical working groups with members from the MoH, implementing NGOs, and the funders played a crucial role in coordinating community health. They were providing technical support to relevant departments of the MoH. For some programmes like for NTDs, the coordination was through the relevant government structures right from the village to the national level.

Nearly all participants referenced insufficient coordination as a limitation to efficient and effective implementation of community health activities. While in some counties, there were coordinated approaches in the implementation of activities, in others, the MoH and NGOs did not always sit together to agree on the modalities (such as the locations) for the implementation, which sometimes led to duplication of services. In some programmes like iCCM, the NGOs were controlling funds meant for implementation, hence, the CHD felt less empowered to monitor the CHW programme activities.

#### Funding of the community health worker programmes

One of the major challenges faced by the South Sudan health sector is inadequate funding and overdependence on donors [23]. Indeed, the main sources of funds for community health programmes were international donors. The donors frequently cited were: United Kingdom’s Department for International Development (DFID) (now Foreign Commonwealth and Development Office (FCDO), United States Agency for international Development (USAID), Canada, and Sweden also (pooled) funding of the HPF programme. Other donors included the Global Fund, the World Bank, the Carter Centre, the African Programme for Onchocerciasis Control, the European Union, the United Nations (UNICEF and WHO), the German Government, the Kingdom of Norway, and the Republic of China. “I am not sure how much contribution the government [South Sudan] has made towards the community health programmes, but the government is providing guidance and leadership” (KI15, Man, UN). Most donors including HPF, channelled funds through NGOs for fear of mismanagement by the government. The participants cited insufficient funding for community health activities compared to the health facility activities. The information from the participants and the HPF reports showed the lack of continuity of some programmes (such as iCCM) once donor funding ended.

### Contextual factors

#### Conflict and insecurity

The participants reiterated that conflicts and insecurity affected the implementation of CHW programmes in the country. Insecurity led to the displacement of communities, looting of medical commodities, and at times death of CHWs. For example, in Nagero County of Western Equatoria State and in Panyijar County in Unity State, insecurity led to the suspension of community health activities. Two participants reported that during the conflict in Panyijar County, the iCCM CBDs continued to provide services to the displaced communities and their hosts.

#### Adverse weather conditions

The participants reported that the rainy season rendered many locations inaccessible. This limited not only the movements of the CHWs and their supervisors, but also hampered the supply of medical commodities.

#### Poor infrastructure

The participants said that poor road infrastructure in some locations hindered movements of CHWs and their supervisors, the distribution of medical commodities, and made referrals difficult. Some participants acknowledged that although CHWs were given cell phones for reporting and keeping in touch with their supervisors, the mobile telephone network coverage was limited in rural areas.

#### Political aspects

The participants mentioned by introducing BHI in 2016, the government has recognised community health as a vehicle to improve access to health services in the communities where there are no health facilities. A few participants mentioned that during the war for independence, the government with support from NGOs used CHWs to provide health education and first aid in the liberated communities.

#### Economic aspects

Some participants mentioned that following the devaluation of the South Sudan currency in 2015, many voluntary CHWs started demanding for financial incentives instead of material incentives. Most CHWs were the breadwinners for their families and therefore needed money to provide for their needs during the economic crisis.

#### Gender aspects

Although many community members were said to prefer female CHWs, participants reported that there were more male CHWs than female ones. This was, according to them, because men are more educated than women. The participants also mentioned that the involvement of women in BHCs was limited despite the requirement that one third of the members should be women.

### Community health worker performance

At the community (end-user) level, many participants mentioned that CHWs promoted community mobilisation and utilisation of some health services. “In our (NGO) programme, the mother-to-mother support groups were an effective tool for behaviour change within the community towards the use of services like for family planning” (KI01, Man, NGO).

Some participants mentioned that CHWs reached areas where no formal health workers from the health facilities could come, and they identified danger signs among mothers and children early, which prompted early referrals. “The communities were testifying to me that iCCM saved the lives of many children and [they] were demanding services to be extended to the other age categories” (KI05, Man, NGO).

The HPF programme reports referenced that community dialogue sessions and advocacy meetings helped to improve uptake of the maternal and child health services. The BHCs contributed to improving water, sanitation, and hygiene activities, and supported health campaigns in the community. Lastly, working with communities helped in organising regular integrated outreach services in remote areas. This improved access to promotive and preventive health services in the community.

### Impact level

Some participants mentioned that CHWs working in disease surveillance have helped the country to progress from being the most guinea worm endemic country in the world to the current zero case reporting. “This is a big achievement on the backdrop of the conflicts and challenges you can think of in this country…” (KI02, Man, MoH).

Most participants reiterated that where there were no health facilities, the iCCM programme increased access to malaria, pneumonia, and diarrhoea treatments in children under five. These might have contributed to the presumed reduction in child deaths related to the three diseases.

## Discussion

Recent CHW programmes in South Sudan were influenced by a variety of factors which were programme related, while others were contextual and health systems related. The programmes used different types of CHWs, and their tasks were mainly in health education and promotion, treatment of some diseases in the children under five, distribution of drugs for NTDs, and facilitating referrals.

The selection of the most suitable community members to become CHWs is vital to the quality and acceptability of health services provided [26, 27]. Similar to the experience in South Sudan, several studies in other settings have established that CHWs are selected based on community membership, social acceptance, gender, knowledge of the culture and languages, personality, past experience, and the level of education [11, 28, 29]. The literacy level in South Sudan is a serious obstacle to the recruitment of CHWs since less than 35% of men and 19.2% of women is literate [30]. Working with communities during the selection process is vital because it ensures that the members understand and accept the work of the CHWs [12, 31]. Selection of CHWs from other communities, in case finding literate candidates is difficult, might not be suitable for the South Sudanese context, where ethnic polarisation and distrust between communities are prevalent. Although the communities led the selection process, we found instances where the selection criteria [20] were not followed. There was also a preference for married women over men and young women over young men since they are deemed to stably reside in the communities. Brown and colleagues, however, advised not to use either age or marital status as selection criteria [12, 27], and it is generally advisable to have a mix of female and male CHWs, depending on the health topics and cultural norms [12, 32, 33].

We found differences in the duration of training sessions for CHWs on similar topics across the counties due to lack of funds and standardised training modules. Elsewhere, studies uncovered variations in the content, quality, length, and training methodologies between CHW programmes [12, 32, 33]. Researchers have reported cases where CHWs received training sessions that were insufficient and of poor quality [29, 33, 34]. Instead, training sessions should be competency-based, to allow the participants to gain the necessary technical and social skills, and the training materials should be in a language understood by the CHWs, preferably with pictographic illustrations [12].

The refresher training sessions were occasional and sometimes did not use of standardised training material. A study from Uganda concluded that regular refresher training sessions for CHWs managing multiple infectious diseases were needed since the initial training sessions were not sufficient to ensure CHW performance [35]. Moreover, international guidelines and experiences elsewhere stipulate that training sessions need to be gender sensitive and responsive since women and men might have different literacy levels and often operate under different social expectations [12, 33]. Some CHW programmes (e.g., for home health promoters and traditional birth attendants) did not have official training curricula. The quality of training, e.g., for CHWs to understand their roles, the services they will offer, and how to provide them, is a critical factor in the success of any CHW programme and requires adequate investment [12, 36].

This study found that the supervision of the CHWs was carried out by supervisors from the community, the health system, and the supporting NGOs. The health facilities and CHDs provided minimal supervision to the CHWs, for example in the iCCM programme. This was partly because health workers perceived supervision of the CHWs as additional work that needs to be incentivised. Similar to our findings, a study done in Guinea, Liberia, and Sierra Leone identified weak links between CHWs and the formal health system as deterrents to effective implementation of community health programmes [37]. Some studies found that during the conflict, the use of community members as CHW supervisors was crucial in increasing the resilience of the iCCM programme [21, 38].

NGOs played an important role in supervising CHWs in the areas where they operated. Supervision should provide opportunities for learning, problem-solving, (community) feedback, and quality assurance. Supervision is an opportunity to assess and strengthen the knowledge and skills of CHWs, thereby improving the quality of service delivery [12, 32, 34, 36, 39, 40]. However, there was inadequate supervision exemplified by infrequent visits due to insecurity, and lack of means of transport. Studies have reported that during conflicts, and where there is insecurity, the frequency of supervision reduces or even stops [21, 41, 42]. A study in Guinea, Liberia, and Sierra Leone also found weak supervision of CHW activities [37]. Additionally, in Rwanda, the infrequent supervisory visits compromised the quality of community health programmes [29].

We found that CHWs received material and financial incentives to facilitate their work, yet incentives were not harmonised across the programmes. Studies have revealed that CHWs are usually given small financial incentives such as honorarium, travel allowance, or other irregular payments but are also motivated by non-financial incentives such as social recognition and prestige, an opportunity to gain knowledge and access to medicines that can benefit their families [32, 33, 43]. Whether CHWs should work as volunteers or should be paid a salary is often under debate [32]. WHO recommends that CHWs should be remunerated based on their task descriptions [12]. Without proper compensation of CHWs, the community health programmes may face high attrition [12, 32, 33, 44].

Our findings show that CHWs’ performance in South Sudan is generally assessed based on the activities conducted and the reports submitted. Generally, a lack of performance management frameworks was found. Performance appraisal of the health workforce is fraught with challenges, however, there is a consensus that the ultimate goal should be to improve motivation and performance of workers for better health outcomes [45, 46]. A recent study proposed a framework for CHW performance measurement and some of the domains include; CHW incentives, supervision and performance appraisal, data use, data reporting, service delivery, quality of services, CHW absenteeism and attrition, community use of services, experience of services, referrals, and trust [47]. Closely linked to supervision is the quality of reported data. Our findings show that the CHWs were reporting on their activities, but the quality of data was regarded as poor.

We found that, despite variation, communities were involved in situation analysis, planning, selection of CHWs, and supervision of community health activities. There is growing consensus that programmes that seek to promote empowerment should involve participation of community members, to offer opportunities for gaining knowledge and skills, confidence, experiences and ability to detect and solve problems [48–52]. Community participation provides people with a sense that members can solve their problems through careful reflection and collective actions [12, 52, 53]. In Ghana, community leaders, trained volunteers and other community members supported health education activities to facilitate skilled birth attendance and contributed land, construction materials, and labour for building health centres [54]. Our findings revealed that in some locations, the BHCs did not exist or were dormant. Given their importance in the BHI strategy, there is need to form or reactivate the committees to ensure ownership and sustainability. Hence, strengthening local governance structures (e.g., the BHCs) and processes, with attention to appropriate representation and inclusion, should be part of the investment in CHW programmes [55].

This study highlights a need for a collaborative relationship between the CHWs and facility staff to ensure accountability, coverage, and quality of care. For example in Mozambique, coordination and communication between the CHWs and the formal health workers enhanced accountability towards the community and the health system [56] and in Uganda, improved communication between CHWs and clinicians through m-health improved the quality of care [57].

Our findings reaffirm that CHW programmes are funded by donors and implemented through NGOs. Kozuki and colleagues reported that there was no funding for iCCM from the national budget. Therefore, once donor funding ceased, the organisation and the structures were left hanging [38]. A study done in Mayendit county in 2017, however, found that when the local authorities have active and responsible roles in the programmes, community engagement is more sustainable [2]. Our findings reaffirm that CHW programmes were fragmented and lacked a standardised regulatory framework. There was inadequate coordination among the community health programmes in the country. Erismann et al., found similar weaknesses in South Sudan and Haiti and recommended establishing and strengthening coordination mechanisms to avoid creating inequalities that might lead to tension and deterioration in social cohesion [2]. Lehmann and others [55] have suggested that harmonising and integrating donor support is an essential building block to the functionality of any CHW programme. Hence, BHI in South Sudan was established to ensure co-ordination and harmonisation of CHW programmes.

Our findings are commensurate with the observation in other fragile and conflict-affected settings where NGOs often create parallel supply chains to ensure the consistent supply of drugs to the NGO supported facilities [21, 58]. Yet, our findings also show inadequate facilities for the storage of medical commodities in the health facilities and at county level. There were also reports of drug misuse leading to stock-outs. The shortage of medical commodities, which are mainly supplied by NGOs, can have consequences beyond the immediate supply, such as a reduction in the trust of CHWs by community members and a decline in utilisation of the services [19, 44, 52]. In Guinea, Liberia, and Sierra Leone during the Ebola outbreak, the supply chain, restriction on movement of the NGO staff due to hostilities, community resistance, and closure of some health facilities hampered service delivery [37]. Other studies in Afghanistan and South Sudan reported that severe weather conditions and insecurity along the roads, poor management, and high distribution costs led to stock-outs of the medical commodities [34, 38, 41].

We identified some contextual factors that affected the previous community programmes and might impact the BHI, such as insecurity, the economy, community, and gender-related issues. Conflicts in Unity and Western Equatoria states led to suspension and even termination of some of the CHW programmes. Some studies from South Sudan demonstrated that in parts of Unity State where there was a conflict, the internally displaced CBDs continued to provide the services to displaced persons and their host communities [2, 38]. In some counties, where weather and security challenges were anticipated, the NGOs and supervisors prepositioned the drugs to last them for longer periods without replenishment. This ensured availability of the drugs and other medical commodities during the crises [38, 41, 59].

While this study did not show a direct relationship between the effects of economic crisis and motivation, the CHWs were reported to prefer financial incentives above material incentives to provide for their families. There were instances in this study where some communities were reported to prefer female CHWs compared to their male counterparts. However, the low level of literacy, especially among women [30], puts them at a disadvantage of being selected as a CHW. Despite the conflicts in the country, the government has made political commitments by introducing the BHI strategy to improve access to healthcare [19, 20]. This, however, remains hampered by a lack of (public) funds and legislative framework to support CHWs who have no formal public health certification.

CHW programmes in non-conflict and conflict affected low and -middle income countries are, to varying degrees, faced with many similar challenges such as inadequate health infrastructure, inadequate training, supervision, motivation, and funding; and securing support from other health care providers and political leaders [60]. However this study shows that the challenges to implementing CHW programmes in conflict affected settings are aggravated and expanded due to insecurity, population displacement, increased shortages of health workers, destruction or theft of infrastructure and medical commodities [61].

The insights from our findings give rise to several policy recommendations. There is a need for the MoH to take a leading role in coordination to ensure ownership and sustainability of the CHW programme. This can be done through policy dialogues, information sharing, participatory decision-making, and resource mobilisation. It is also essential for policymakers to design an incentive structure that will not create a financial burden to the government and the communities. To ensure a strong and functional CHW programme, there is a need for a collaborative relationship between the CHWs and the health facility staff. This will facilitate supervision, enhance accountability, supply of medical commodities and referral of clients. Supervision should be frequent, regular and use standard supervision guidelines with clearly defined objectives to reinforce knowledge, skills, competencies, and motivation. Lastly, a minimum quality of training can be assured through using set guidelines and/or curricula based on the context and education level of the CHWs and the type of work they are expected to perform.

### Study limitations

This study was subject to several limitations. The author’s roles in providing operational research insights to the HPF consortium and their interplay with research participants, who have a stake in the success of and (continued) funding for CHW programmes, might have introduced certain participant biases. Participants were recruited through the first author’s and HPF’s network, which may have led to some degree of respondent bias. Furthermore, the study focus was primarily on participants who were either policymakers or programme implementers. Given the dependence on (future) donor support for the implementation of CHW initiatives, this could have resulted in them being less critical about CHW programmes in general and particularly the programmes they were responsible for. The study, however, made a conscious effort to cross-validate and triangulate information from stakeholders with different interests in the matter, as the overview of research participants shows, and mostly concerns programmes that were not funded by HPF. The study was carried out at the onset of BHI implementation; thus, some aspects of the strategy may not have been clear to both the participants and the researchers and certain policy details may have shifted.

The community activities presented in the HPF reports were only those under the programme contractual obligations. The study did not capture the views of CHWs or community members directly, and, therefore, cannot be taken to represent their experiences. As the BHI is being scaled up, future research should include CHWs to learn from their experiences and strengthen the evidence for the relevance of factors influencing CHW performance. This includes due attention to the power context in which both Boma health workers and citizens operate, influencing CHWs’ ability to deliver services and the citizen’s ability to access them.

## Conclusions

Despite rampant conflicts in South Sudan, a variety of CHW programmes have been implemented in recent years. Our findings provide insights into the factors influencing these programmes in South Sudan as learnings for the implementation of BHI and the design and implementation of CHW programmes in other low resource, conflict-affected settings. Many of the factors were related to the way the programmes were designed, in terms of CHW tasks, human resources management, community participation, as well as aspects of quality assurance. Others were related to governance, coordination, and funding of CHW programmes. Although outside the programmes’ control, conflict, adverse weather conditions and poor infrastructure affected the programmes. It is important that policy makers evaluate this evidence to enable the successful implementation of the BHI strategy in South Sudan, thereby improving access to and quality of health services in the country, contributing to the universal health coverage. For effective implementation of the BHI strategy and CHW programmes in comparable settings, there is a need for robust coordination and cooperation among stakeholders.

## Data Availability

Not available.

## Abbreviations

AIDS: Acquired Immune Deficiency Syndrome
BHC: Boma Health Committee
BHI: Boma Health Initiative
CBD: Community Based Distributor
CDD: Community Drug Distributor
CHD: County Health Department
CHW: Community Health Worker
DFID: Department for International Development
FCDO: Foreign Commonwealth and Development Office
HIV: Human Immune Deficiency Virus
HPF: Health Pooled Fund
iCCM: Integrated Community Case Management
MoH: Ministry of Health
NGO: Non-Governmental Organisation
NTD: Neglected Tropical Diseases
UN: United Nations
UNICEF: United Nations Children’s Fund
USAID: United States Agency for International Development
WHO: World Health Organization

## Footnotes

1 ‘Boma’ is the lowest administrative unit of local government in South Sudan.

2 A payam is the second-lowest administrative division, below counties, in South Sudan.

3 iCCM was targeting treatment of malaria, pneumonia, diarrhoea, and screening for malnutrition for children the under five years of age.

4 Nine months trained CHW is a paid and health facility (PHCU) based worker responsible for provision of curative, preventive, and promotive services, including maternal health and health information management.
